# Implication of Neutrophils Extracellular Traps in the Pathogenesis of SARS-CoV-2 pneumonia

**DOI:** 10.3390/biomedicines10102638

**Published:** 2022-10-20

**Authors:** Patricia Pérez-Guerrero, Francisco Illanes-Álvarez, Denisse Márquez-Ruiz, Irene Campaña-Gómez, Sara Cuesta-Sancho, Mercedes Márquez-Coello, José-Antonio Girón-González

**Affiliations:** 1 Servicio de Medicina Interna, Hospital Universitario Puerta del Mar, Facultad de Medicina, Universidad de Cádiz, Instituto para la Investigación e Innovación Biomédica de Cádiz (INiBICA), 11009 Cádiz, Spain; 2 Departamento de Inmunología, Facultad de Medicina, Universidad de Valladolid, 47003 Valladolid, Spain

**Keywords:** NETs, polymorphonuclear neutrophils, vascular cell adhesion molecule 1, vascular growth endothelial factor, E-selectin, P-selectin, CXC chemokine ligand 4/Platelet factor 4, COVID-19, SARS-CoV-2

## Abstract

Peripheral blood polymorphonuclear neutrophils (PMNs) forming extracellular traps (NETs), as well as endothelial- and platelet-derived parameters, have been analyzed in patients with SARS-CoV-2 pneumonia, and their prognostic role has been evaluated. Eighty-seven consecutive patients hospitalized with SARS-CoV-2 pneumonia were prospectively selected. A sample of 30 healthy individuals served as the control group. Clinical and oxygenation (oxygen saturation to fraction of inspired oxygen ratio—SpO^2^/FiO^2^) characteristics and PMNs forming NETs, serum levels of myeloperoxidase, E-selectin, vascular cell adhesion molecule 1—VCAM1—vascular endothelial growth factor, P-selectin, platelet factor 4 and plasma concentrations of D-dimer were evaluated at hospital admission, at discharge and 14 days after discharge. Intensive care unit admission or death was the primary composite endpoint. Patients showed a higher number of PMNs forming NETs than healthy controls. The absolute number of PMNs forming NETs was inversely correlated with oxygen status (SpO^2^/FiO^2^) and positively with inflammatory (C-reactive protein, ferritin) markers and VCAM1. A decrease in, but not a normalization of NETs and endothelial-derived parameters was observed in patients who survived. In conclusion, the formation of NETs runs parallel to that of other inflammatory and endothelial activation markers, and is inverse to the oxygenation parameters, supporting a pathogenic role for PMNs in this entity.

## 1. Introduction

Polymorphonuclear neutrophils (PMNs) play a role in the pathogenesis of COVID-19, as has been supported by the following observations: (a) Lung samples obtained after necropsy of patients with SARS-CoV-2 pneumonia have shown the presence of PMNs in the airway (even occluding bronchioles and alveoli), in interstitial tissue (usually simultaneously with macrophagic but not lymphocytic infiltrate) and microthrombi (in conjunction with platelets and fibrin) in arterioles [1,2,3]. (b) In bronchoalveolar lavage samples from patients, the predominant cells are PMNs [4]. In addition, an increase in PMNs has been observed in individuals with severe COVID-19 [5]. (c) A prominent feature of COVID-19 is multiorgan inflammation with vessel walls containing neutrophils [6]. (d) An increase in the number of PMNs (or the neutrophil to lymphocyte ratio—NLR) is associated with greater severity and worse prognosis in patients with COVID-19 [7,8].

In infections, neutrophils are one of the first lines of defense, exerting their action through phagocytosis, production of oxygen radicals, secretion of proteolytic enzymes and cytokines or formation of NETs (neutrophil extracellular traps). NETosis is a cell death mechanism in which neutrophils release NETs to the extracellular space. NETs are chromatin networks containing histones, microbicidal peptides or oxidative enzymes [9]. This is a multistep process: enzymes from cytoplasmic granules translocate to the nucleus and decondense chromatin, which is followed by a rupture of the nuclear and granule membranes, and finally, after cytolysis, NETs are released [9]. NETs are highly adherent and capture extracellular microbes such as bacteria, fungi or viruses, stimulating their elimination [10]. An acute and controlled NETosis is an efficient defense mechanism since it prevents collateral tissue damage, releasing antimicrobial molecules and reducing the toxicity attributable to proteases; however, an uncontrolled NETosis contributes to propagating the inflammatory response and favors microvascular thrombosis [11]. The role of NETs has been demonstrated in sepsis, in which significant inflammatory response and thrombosis occurs [12]. It has been observed that the expression of genes involved in NET formation is increased in patients with COVID-19 [5], suggesting that they may contribute locally to lung injury.

The objectives of this work were dual: (1) Analyze peripheral blood NETs (by flow cytometry), as well as molecules released by PMNs (myeloperoxidase—MPO), or implicated in the endothelium (E-selectin, vascular cell adhesion molecule 1—VCAM1—vascular endothelial growth factor—VEGF) and platelets (P-selectin, CXC chemokine ligand 4/Platelet factor 4—PF4) activation in patients with SARS-CoV-2 pneumonia, at hospital admission and after resolution. (2) Characterize the prognostic role of NETs in SARS-CoV-2 pneumonia, while also considering the National Early Warning Score 2—NEWS2 [13]—NLR [14], and C-reactive protein [15], ferritin [16] and D-dimer [17] levels.

## 2. Materials and Methods

### 2.1. Patients

Consecutive patients hospitalized at University Hospital Puerta del Mar (Cadiz, Spain) between 1 February 2021 and 30 June 2021 were eligible for inclusion if they were 18 years of age or older, had a confirmed COVID-19 infection and had radiographic evidence of pulmonary infiltrates. 

Exclusion criteria were: (1) Patients with concomitant microbes that could be implicated in the etiology of pneumonia. (2) Patients with confirmed SARS-CoV-2 infection who had been admitted for other reasons and had no signs or symptoms of COVID-19. (3) Patients with systemic autoimmune diseases due to the expected elevation of NETs in them. (4) Patients with acute coronary or cerebrovascular disease, as well as acute lower extremity ischemia, in the two weeks prior to COVID-19 pneumonia due to expected elevation of platelet or endothelial activation parameters. (5) Renal failure (glomerular filtration rate less than 25 mL/min) due to its influence on the serum concentration of certain biomarkers. (6) Patients who did not accept entering the study or the follow-up.

The sample size was derived from all eligible consecutive hospitalized patients during the study period.

Thirty age- and gender-matched healthy individuals were recruited for comparison of PMNs-, endothelial- and platelet-related parameters.

### 2.2. Study Design

This was a prospective observational study of hospitalized patients with SARS-CoV-2 pneumonia. The conduct of the research and the dynamics of the study were carried out according with the Strengthening of the Observational Studies Report in Epidemiology Guidelines (STROBE) [18].

The primary composite endpoint was transfer to the intensive care unit (ICU) from a general medical unit due to a need for mechanical ventilation, or in-hospital all-cause mortality.

The severity of the patients was classified according to the NEWS2 score (National Early Warning Score 2). NEWS2 score includes respiratory rate, need of supplemental oxygen, oxygen saturation, hypercapnic respiratory failure, systolic blood pressure, pulse, temperature and level of consciousness. A NEWS2 index > 6 is considered the appropriate cut-off point based on its sensitivity and specificity [13,19]. 

All patients were treated with a uniform protocol developed by consensus of Internal Medicine Department Physicians. Supplemental oxygen and supportive care were indicated on a case-by-case basis. Enoxaparin (at a prophylactic dose, according to the individual’s weight) was indicated in all patients, with the exception of those who needed full anticoagulation due to previous or posterior comorbidities. Patients with an oxygen saturation (measured by pulse oximetry) to inspired oxygen fraction ratio (SpO^2^/FiO^2^) of ≤315, and those with an elevation of acute phase reactants (serum ferritin concentration of >1000 ng/mL or C-reactive protein (CRP) of >100 mg/dL), started treatment with dexamethasone once daily for up to 10 days or until hospital discharge. In those patients whose hypoxemia did not improve after corticosteroids, tocilizumab was added. 

Exclusion criteria for the use of corticosteroids or tocilizumab were coexistent infection other than COVID-19; history of severe allergic reactions to monoclonal antibodies; less than 500 neutrophils/mm^3^ or less than 50,000 platelets/mm^3^; symptomatic gastrointestinal tract condition that might predispose patients to intestinal perforation; or severe impairment of hematologic, renal, or hepatic function.

Those patients who required invasive mechanical ventilation for severe acute respiratory distress syndrome (ARDS) (respiratory rate of 30 or more breaths per minute, required FiO_2_ at 80% or more to maintain a SpO^2^ level of 90%, or a PaO_2_/FiO_2_ ratio of less than 100 mm Hg) and those who had good performance status prior to the admission, were admitted in the ICU. 

### 2.3. Data Collection

SARS-CoV-2 was confirmed by real-time RT-PCR in a nasopharyngeal, sputum or bronchoalveolar sample. Other respiratory viruses including influenza A and B, respiratory syncytial, parainfluenza and adenovirus were excluded by film array, according to the judgment of the responsible clinician. Sputum or endotracheal aspirates cultures were obtained when available at admission for identification of possible concomitant causative bacteria or fungi. 

Data were obtained from electronic medical records. Clinical data included age, sex, comorbidities, symptoms on admission, respiratory status and time from disease onset. SpO^2^/FiO^2^ is considered as a surrogate marker for partial pressure of oxygen to fraction of inspired oxygen ratio and a predictor of the development of ARDS [20]. A good relationship between SpO^2^/FiO^2^ and the ratio of the partial pressure of arterial oxygen to the fraction of inspired oxygen (PaO^2^/FiO^2^) has been observed [21]. Chest x-rays were performed in all patients. 

Laboratory values at admission (T0), at discharge (TDi) and 14 days after discharge (T+14Di) included:

(1) General parameters: neutrophil count, lymphocyte count and neutrophil to lymphocyte ratio—NLR—and coagulation parameters including D-dimer (DD), CRP and ferritin. 

(2) The formation of NETs by PMNs was determined. DNAse I levels, as main catabolic enzyme of NETs, were also analyzed. Myeloperoxidase—MPO—as another PMN-derived molecule, was determined. 

(3) Parameters implicated in endothelial (E-selectin, VCAM1, VEGF) and platelet (P-selectin, PF4) activation. 

### 2.4. Laboratory Determinations

To determine PMNs forming NETs (CD10+CD11b+Hist3cit+MPO+), flow cytometry was conducted in whole peripheral blood, collected in ethylenediaminetetraacetic (EDTA) tubes. For PMN characterization, membrane expression of CD10 (clone HI10a, BD Biosciences, San Jose, CA, USA) and CD11b (clone ICRF44, BD Biosciences, San Jose, CA, USA) was analyzed. Cells were fixed and permeabilized with BD Cytofix/Cytoperm™ Fixation/Permeabilization solution Kit with BD GolgiPlug™ (BD Biosciences, San Jose, CA, USA), following product recommendations. Then, cells were incubated with human anti-citrullinated histone 3 (Abcam, Cambridge, UK). An Alexa Fluor 488 (Abcam, Cambridge, UK) was used as a secondary antibody. IgG isotypes were used as negative controls. 

The blood samples, collected in sterile EDTA/Vacutainer tubes, were centrifuged (3500× *g*, 15 min) and the plasma/serum, respectively, stored at −70 °C in pyrogen-free polyethylene tubes (Biofreeze, Costar, Washington, DC, USA) until the time of analysis of the plasma/serum concentration of the molecules. The concentrations of MPO and PF4 were determined by ELISA (R&D, Minneapolis, MN, USA). The concentrations of P- and E-selectins, VCAM1 and VEGF were measured by Luminex Human Discovery Assay (R&D, Minneapolis, MN, USA). The DNase I was measured using the Human DNase-I (deoxyribonuclease I) ELISA Kit (Biomatik, Wilmington, DE, USA) [22].

### 2.5. Statistical Analysis

Median and interquartile ranges were used for quantitative variables, absolute numbers and percentages for qualitative variables. Differences in the values of parameters studied between T0, TDi and T+14Di were analyzed by Wilcoxon test. A bivariate analysis was performed to compare clinical and laboratory parameters between the group of healthy controls and patients, and, among patients, between survivors and non-survivors or ICU-admitted patients. The chi-square test and Fisher’s exact test were used for comparison of categorical variables and the Mann–Whitney U test was used for continuous variables. Variables with *p* values of <0.1 in the bivariate analysis between the group of survivors and non-survivors or ICU-admitted patients were included in the multivariate model. Cox regression was utilized to identify independent variables associated with the primary outcome: the number of days from admission to hospitalization discharge or ICU admission/death. The Kaplan–Meier method and log-rank *p* value were used to compare time-to-discharge alive from hospital versus those who died or needed ICU admission. 

Statistical analysis was performed with SPSS for Windows version 22.0 (SPSS, Inc., Chicago, IL, USA), considering a value of *p* < 0.05 to be statistically significant.

## 3. Results

A total of 87 patients with COVID-19 pneumonia were admitted at the hospital and selected for the study (Table 1). 

Increased values of NLR and levels of D-dimer, CRP and ferritin were detected in patients compared with healthy controls.

### 3.1. PMNs-Related Parameters and Endothelial- and Platelets-Derived Variables in Patients and Controls

The absolute number and percentage of PMNs that formed NETs was significantly higher in COVID-19 patients. Serum MPO levels were similar in patients and controls, as well as those of the enzyme implicated in the elimination of NETs, DNAse I (Table 2). 

VCAM1 and VEGF, two parameters associated with endothelial activation, were significantly higher in individuals with COVID-19. A significant decrease in serum levels of P-selectin and PF4 was observed in patients with SARS-CoV-2 pneumonia (Table 2).

**Figure 1:**
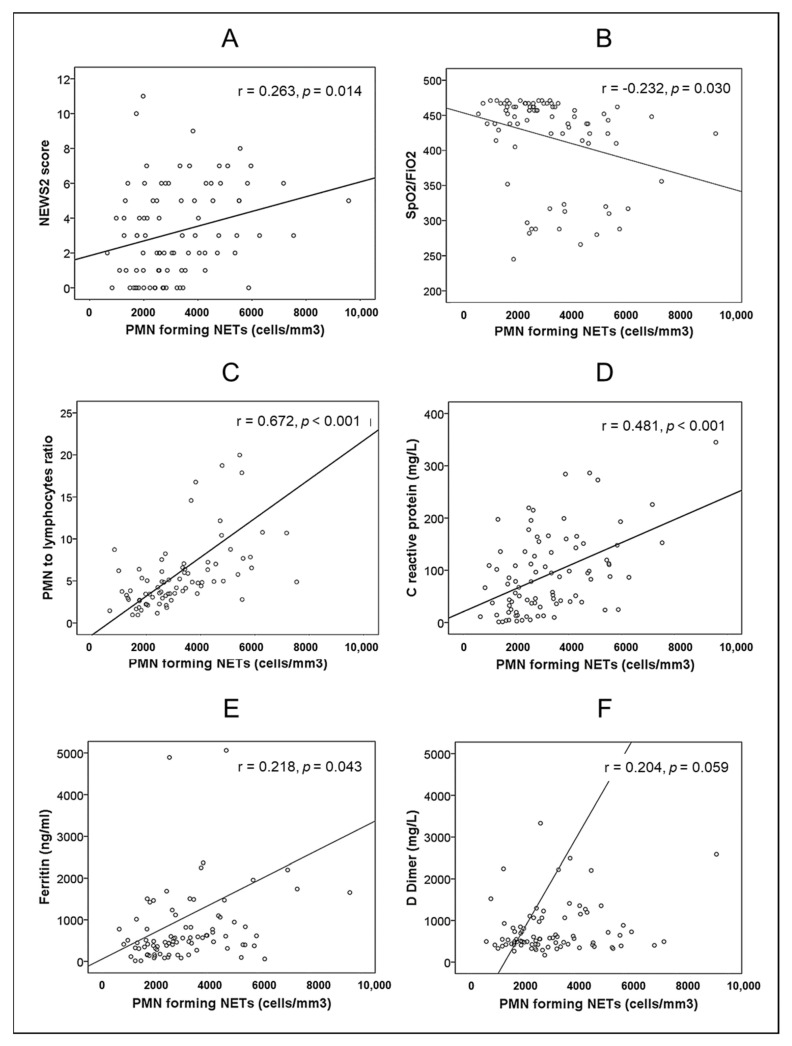
Bivariate correlations between absolute number of PMNs forming NETs and: (**A**) NEWS2 score. (**B**) SpO^2^/FiO^2^. (**C**) PMN to lymphocyte ratio. (**D**) Serum concentration of C-reactive protein. (**E**) Serum concentration of ferritin. (**F**) Plasma concentration of D-dimer.

A significant correlation was observed between the absolute number of PMNs forming NETs and classical parameters implicated in the prognosis of these patients: NEWS2 score, SpO_2_/FiO_2_, NLR and serum levels of CRP or ferritin, although not with D-dimer (Figure 1). 

**Figure 2:**
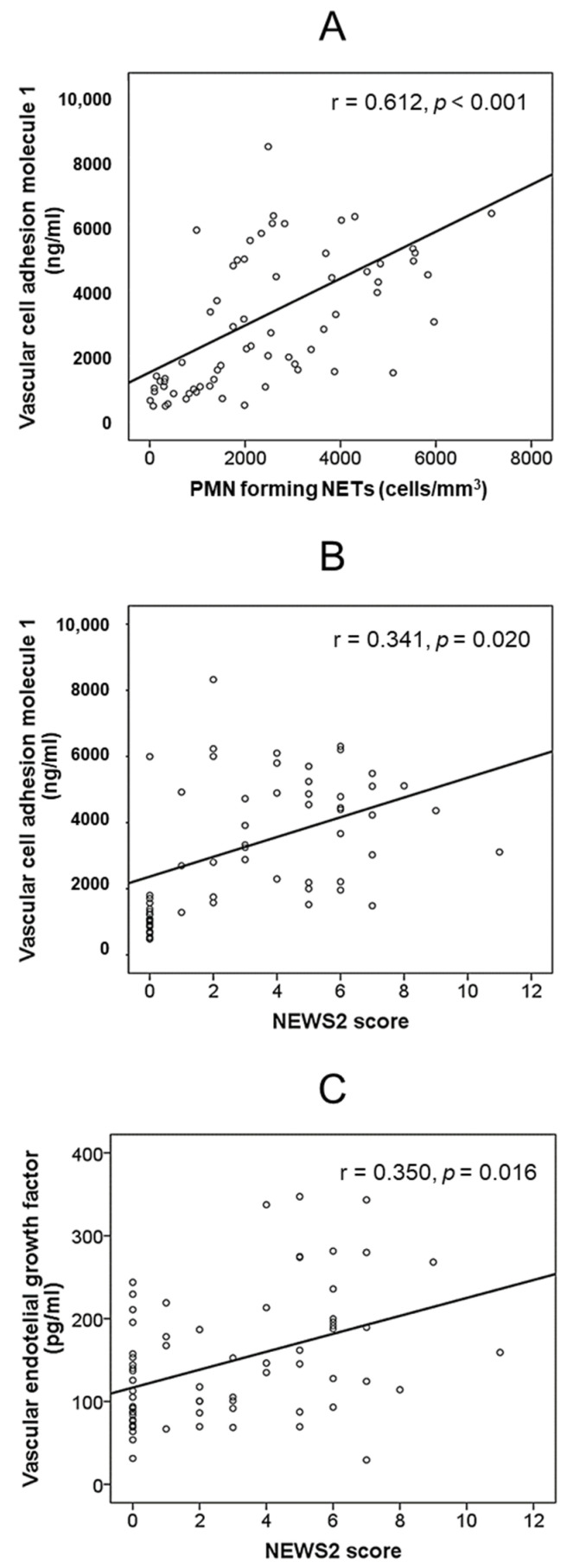
Bivariate correlations between: (**A**) Absolute number of PMNs forming NETs and serum concentration of vascular cell adhesion molecule 1. (**B**) NEWS2 score and serum concentration of vascular cell adhesion molecule 1. (**C**) NEWS2 score and serum concentration of vascular endothelial growth factor.

The absolute number of PMNs forming NETs and the concentration of VCAM1 were significantly correlated as well (r = 0.612, *p* < 0.001). No significant correlation was detected between the absolute number of PMNs forming NETs and other endothelial- and platelet-derived parameters (data not shown). NEWS2 score correlated with VCAM1 levels (r = 0.341, *p* = 0.020) and VEGF (r = 0.350, *p* = 0.016), but not with platelet-derived molecules (Figure 2).

### 3.2. Outcomes of Patients during Hospitalization

After admission, 72 patients (82.8%) received corticosteroids and 29 (33.3%), tocilizumab. Nonetheless, seven patients needed ICU admission (8%) by progressive respiratory failure and, eventually, died. Baseline characteristics of patients are shown by outcome in Table 3.

Non-surviving patients were diabetic more often and had a significantly higher punctuation in NEWS2 score and lower SpO_2_/FiO_2_. 

**Figure 3:**
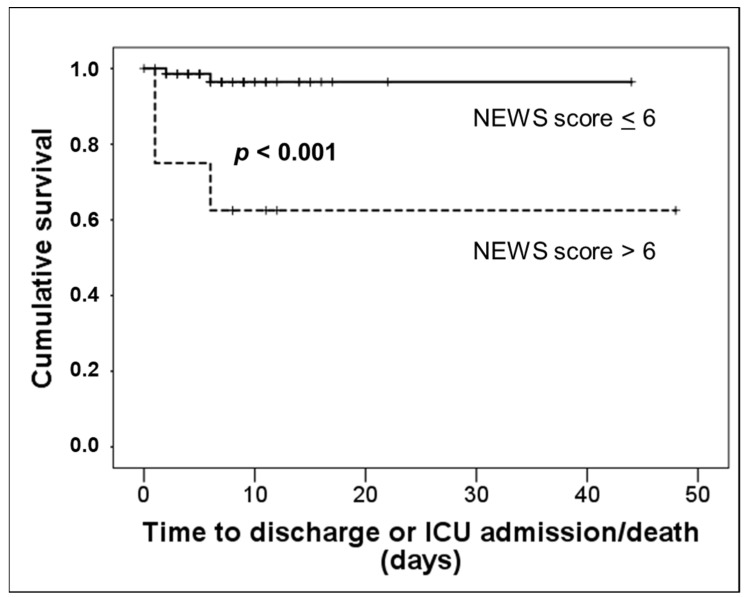
Kaplan–Meier survival curves of patients with pneumonia by SARS-CoV-2 as a function of a NEWS2 score ≤ 6 (continuous line) or >6 (dashed line).

Multivariate analysis by Cox regression demonstrated that the only independent factor associated with survival was the NEWS2 score (Exp (B) 1.389, confidence interval 95% 1.005–1.920, *p* = 0.046). Kaplan–Meier survival curves as a function of a NEWS2 score ≤ 6 or >6 are shown in Figure 3.

Surviving patients were followed up. The median time of hospitalization was 7.0 (4.0–9.0) days. Studied variables were analyzed at hospital discharge and 14 days after it. The values of these parameters are shown in Table 4.

SpO_2_/FiO_2_ had significantly improved at discharge compared to admission. Additionally, both CRP and DD levels, but not ferritin, had normalized at discharge or 14 days after discharge.

Although PMNs forming NETs and VCAM1 decreased significantly, the reported levels were still elevated compared to healthy controls at discharge and 14 days after (*p* < 0.001 respectively to healthy controls in each case). Another endothelial-derived parameter, VEGF, normalized its serum concentrations 14 days after discharge. In contrast, DNAse-I increased significantly during follow-up. A significant elevation of platelet activation parameter values was observed during hospitalization, diminishing to baseline levels in the determination made 14 days after discharge.

## 4. Discussion

We analyzed the role of PMNs in patients with SARS-CoV-2 pneumonia, focusing on their ability to produce NETs. An increased number of PMNs forming NETs was demonstrated in these patients.

Most studies about the pathogenesis of COVID-19 have focused on the role of macrophages [23,24], paying little attention to PMNs. However, there is intense PMN infiltration in the alveoli and the pulmonary interstitial space of patients with SARS-CoV-2 pneumonia [1], and the predominant cells in the bronchoalveolar lavage are also PMNs [3,4], as well as in the microvascular thrombi in the pulmonary capillaries, in conjunction with platelets [3]. 

One of the possible mechanisms by which PMNs cause tissue damage and thrombosis is the formation of NETs. An uncontrolled NETosis may contribute to propagate inflammatory response [11]. Compared to healthy subjects, patients with COVID-19 have elevated serum levels of surrogate markers indicative of the existence of NETs, such as free-DNA, MPO-DNA complexes, and citrullinated histone H3 [25,26,27]. The values of these indirect indices of NETs are significantly higher in patients with worse gasometric measures [25,26,28,29].

In this study, we have used a procedure to directly detect the formation of NETs by PMNs, demonstrating increased levels in patients with SARS-CoV-2 pneumonia. The level of the other mediator of PMNs, MPO, did not follow the same pattern; indeed, its serum concentration was similar to that of healthy controls. Shrivastava et al. demonstrated elevated MPO in only a subgroup of these patients [30]. Our findings suggest that NET formation and MPO secretion could follow different pathways in COVID-19. It is possible to speculate that the MPO secreted by PMNs (and possibly other enzymes) would be trapped in these neutrophilic networks, with no detectable increase in peripheral blood.

The stimuli associated with the production of NETs include SARS-CoV-2 itself [31], antigens associated with cell lesions [32,33], activated endothelial cells [34] and proinflammatory cytokines [10,35,36]. In this sense, the significant correlation detected in the present work between the NET count and the parameters indicative of inflammation studied (CRP, ferritin) was remarkable. Even more interesting, the number of PMNs forming NETs was directly correlated with the widely validated NEWS2 prognostic scale and inversely correlated with oxygenation status (determined by the SatO^2^/FiO^2^ ratio).

Varga et al. have revealed the accumulation of inflammatory cells associated with endothelium in COVID-19 [6]. Activated endothelial cells release leukocyte adhesion molecules, pro-inflammatory cytokines, and chemokines in COVID-19 individuals [37]. Endothelial adhesion molecules that mediate vascular inflammation include, among others, E-selectin and VCAM1 [37]. Indeed, serum VCAM1 concentration, as well as that of the other endothelium-derived molecule studied, VEGF, has been linked with disease severity in COVID-19 patients [38,39] and this was also detected in our study. The existing correlation between the number of NETs and VCAM1 suggests a reciprocal influence of PMNs towards the triggering of microcirculation alterations and of endothelial activation in the continued generation of NETs. 

There was no correlation between NET count and platelet activation (PF4, P-selectin) or coagulation (D-dimers) parameters, advocating against a possible direct influence of NETs in the activation of the coagulation cascade. Along the same line, other studies have not shown a relationship between the development of pulmonary thromboembolism and the quantification of NET surrogate markers in peripheral blood [28].

Analysis of the outcomes of the studied parameters in surviving patients was interesting. PMNs forming NETs were significantly lower at hospital discharge of surviving patients and continued to decrease thereafter. On the contrary, the activity of the enzyme responsible for its dissolution, DNAse I, increased with respect to the moment of admission, suggesting a higher activity to eliminate NETs. 

The RECOVERY study has demonstrated the usefulness of dexamethasone in the treatment of COVID-19 [40]. This drug was administered to 81.5% of the patients in this study. Dexamethasone inhibits the formation of NETs in vitro [41]. The effect of tocilizumab on NETs formation is not known.

The outcomes of the PMNs forming NETs were parallel to that followed by the parameters indicating inflammation (such as CRP and ferritin) and vascular activation (VCAM1, VEGF) and inverse to that of the oxygenation parameters (SpO^2^/FiO^2^). In any case, it is interesting that PMNs forming NETs and VCAM1 values 14 days after hospital discharge remained higher than those of healthy controls. The increase in VCAM1 provides an explanatory basis for the thrombotic phenomena detected in these patients, mainly, although not exclusively, during the acute period of the disease [42]. Also, persistent neutrophilic and endothelial activation could be the basis of the alterations that characterize the long-COVID developed by some of the patients [43]. The analysis of these parameters in patients with persistent COVID-19 is required to clarify this aspect.

This work has several limitations. First, although a direct analysis of NETs has been carried out, their influence on endothelium or platelets through in vitro studies has not been evaluated. Second, despite the demonstration that NETs have a role in COVID-19, their prognostic influence has not been proven. A more robust prognostic index, the NEWS2 score, was the only variable associated with survival. 

## 5. Conclusions

An increase in the formation of NETs is evident in individuals with SARS-CoV-2 pneumonia from the first moments of the disease. Their formation runs parallel to that of other inflammatory and endothelial activation markers, and is inverse to the oxygenation parameters of individuals, supporting a pathogenic role for PMNs in this entity.

## Figures and Tables

**Table 1:** Baseline characteristics of healthy controls and patients hospitalized by COVID-19 pneumonia.

Variable		Healthy Controls (*n* = 30)	Patients with SARS-CoV-2 Pneumonia (*n* = 87)	*p*
Age (years)		56 (48–70)	56 (44–68)	0.568
Sex male (*n*,%)		18 (60)	54 (62)	1.000
Comorbidity				
	Arterial hypertension (*n*,%)	0 (0)	33 (38)	
	Diabetes mellitus (*n*,%)	0 (0)	18 (21)	
	Obesity (body mass index > 30 kg/m^2^) (*n*,%)	0 (0)	22 (25)	
Time from first symptom to admission (days)			9 (7–11)	
NEWS2 score				
	NEWS2 (points)		3 (1–5)	
	NEWS2 > 6 (*n*,%)		10 (12)	
SpO_2_/FiO_2_			448 (405–462)	
Polymorphonuclear neutrophils (cells/mm^3^)		3575 (2860–4250)	3990 (2810–5650)	0.168
Lymphocytes (cells/mm^3^)		1990 (1608–2418)	850 (660–1205)	<0.001
Neutrophil to lymphocyte ratio		1.9 (1.5–2.4)	4.8 (3.0–6.6)	<0.001
D-Dimer (mg/L)		382 (273–466)	547 (420–996)	<0.001
C-reactive protein (mg/L)		2 (1–4)	79 (33–136)	<0.001
Ferritin (ng/mL)		78 (50–116)	484 (313–1014)	<0.001

Results are shown as median (interquartile range) or as number (percentage).

**Table 2:** PMNs-related parameters, and endothelial- and platelet-derived variables in control and patients at baseline.

Variable		Healthy Controls (*n* = 30)	Patients with Pneumonia by SARS-CoV-2 (*n* = 87)	*p*
**PMN-derived parameters**				
	PMNs forming NETs (cells/mm^3^)	324 (134–803)	2751 (1983–4261)	<0.001
	PMNs forming NETs (% of PMNs)	10 (4–25)	75 (65–89)	<0.001
	Myeloperoxidase (ng/mL)	680 (499–1233)	875 (490–1498)	0.373
	DNAse I (ng/mL)	8.8 (5.6–12.9)	6.1 (2.4–12.4)	0.079
**Endothelial-derived molecules**				
	E-selectin (ng/mL)	31 (21–45)	33 (20–49)	0.765
	Vascular cell adhesion molecule 1 (ng/mL)	989 (673–1224)	4363 (2212–5487)	<0.001
	Vascular endothelial growth factor (pg/mL)	105 (74–149)	146 (93–213)	0.036
**Platelet-derived molecules**				
	Platelet factor 4 (ng/mL)	9589 (7910–12,223)	7990 (6190–9697)	0.006
	P-selectin (ng/mL)	54 (43–58)	46 (32–58)	0.043

Results are shown as median (interquartile range).

**Table 3:** Baseline characteristics of patients with pneumonia by SARS-CoV-2, grouped by outcome.

Variable		Surviving Patients (*n* = 80)	Non-Surviving Patients (*n* = 7)	*p*
Age (years)		56 (44–68)	62 (49–68)	0.353
Sex male (*n*,%)		50 (63)	4 (57)	1.000
Comorbidity				
	Arterial hypertension (*n*,%)	29 (36)	4 (57)	0.419
	Diabetes mellitus (*n*,%)	14 (17)	4 (57)	0.031
	Obesity (body mass index > 30 kg/m^2^) (*n*,%)	19 (24)	3 (43)	0.362
Time from first symptom to admission (days)		9 (7–11)	8 (7–11)	0.437
NEWS2 score				
	NEWS2 (points)	2 (1–5)	7 (6–7)	0.001
	NEWS2 > 6 (*n*,%)	6 (8)	4 (57)	0.003
SpO^2^/FiO^2^		448 (417–466)	310 (282–410)	0.002
C-reactive protein (mg/L)		79 (30–136)	88 (42–165)	0.657
Ferritin (ng/mL)		481 (313–997)	591 (282–5062)	0.492
D-Dimer (mg/L)		533 (415–973)	734 (465–1066)	0.533
Polymorphonuclear neutrophils (cells/mm^3^)		3845 (2810–5585)	5350 (4360–6340)	0.180
Lymphocytes (cells/mm^3^)		855 (638–1208)	820 (750–1100)	0.919
Neutrophil to lymphocyte ratio		4.2 (2.8–6.5)	5.0 (4.4–7.7)	0.261
PMN forming NETs (cells/mm^3^)		2725 (1974–4013)	4018 (2105–4791)	0.165
PMN forming NETs (% of PMNs)		76 (65–89)	69 (34–89)	0.731
Myeloperoxidase (ng/mL)		869 (500–1500)	870 (490–1452)	0.985
DNAse I (ng/mL)		6.1 (2.5–12.4)	6.1 (2.4–9.8)	1.000
E-selectin (ng/mL)		32 (19–47)	45 (30–61)	0.086
Vascular cell adhesion molecule 1 (ng/mL)		3791 (2049–5162)	5112 (4230–6101)	0.118
Vascular endothelial growth factor (pg/mL)		146 (88–195)	213 (114–281)	0.134
Platelet factor 4 (ng/mL)		7993 (6198–9697)	7885 (6120–12,200)	0.450
P-selectin (ng/mL)		45 (31–59)	47 (34–48)	0.826

Results are shown as median (interquartile range) or as number (percentage).

**Table 4:** Outcomes in surviving patients with pneumonia by SARS-CoV-2 (*n* = 80).

Variable	At Admission (T0)	At Discharge (Tdi)	14 Days after Discharge (T+14di)	*p* T0 vs. Tdi	*p* T0 vs. 14Tdi	*p* Tdi vs. T14di
SpO_2_FiO_2_	448 (417–466)	465 (457–467)	462 (457–467)	<0.001	<0.001	0.251
C-reactive protein (mg/L)	79 (30–136)	5 (2–8)	1 (0–3)	<0.001	<0.001	<0.001
Ferritin (ng/mL)	481 (313–997)	446 (277–930)	201 (84–348)	0.374	<0.001	<0.001
D-Dimer (mg/L)	533 (415–973)	457 (299–736)	334 (250–791)	<0.001	<0.001	0.085
Neutrophil to lymphocyte ratio	4.2 (2.8–6.5)	2.3 (1.5–3.5)	2.1 (1.4–2.8)	<0.001	<0.001	0.351
PMNs forming NETs (cells/mm^3^)	2725 (1974–4013)	1270 (590–2305)	702 (270–1305)	<0.001	<0.001	0.003
Myeloperoxidase (ng/mL)	869 (500–1500)	605 (456–1475)	340 (258–500)	0.278	<0.001	<0.001
DNAse I (ng/mL)	6.1 (2.5–12.4)	23.9 (11.9–35.9)	13.1 (7.1–23.8)	<0.001	<0.001	<0.001
E-selectin (ng/mL)	32 (19–47)	30 (21–42)	35 (22–46)	0.350	0.677	0.128
Vascular cell adhesion molecule 1 (ng/mL)	3791 (2049–5162)	2023 (1224–2582)	1390 (1070–2045)	<0.001	<0.001	<0.001
Vascular endotelial growth factor (pg/mL)	146 (88–195)	132 (92–285)	97 (64–125)	0.009	<0.001	<0.001
Platelet factor 4 (ng/mL)	7993 (6198–9697)	10,793 (7128–14,524)	7423 (4731–9611)	<0.001	<0.001	0.053
P-selectin (ng/mL)	45 (31–59)	58 (49–59)	49 (42–58)	<0.001	0.066	<0.001

Results are shown as median (interquartile range).

## Data Availability

All data generated or analyzed during this study are included in this published article.
